# Bis(di-2-pyridyl­amine-κ^2^ *N* ^2^,*N* ^2′^)palladium(II) bis­(thio­cyanate)

**DOI:** 10.1107/S1600536812033958

**Published:** 2012-08-04

**Authors:** Kwang Ha

**Affiliations:** a School of Applied Chemical Engineering, The Research Institute of Catalysis, Chonnam National University, Gwangju 500-757, Republic of Korea

## Abstract

The Pd^II^ atom of the title salt, [Pd(C_10_H_9_N_3_)_2_](NCS)_2_, lies on a center of inversion and exists in a square-planar environment defined by the four pyridine N atoms derived from the two chelating di-2-pyridyl­amine (dpa) ligands. The chelate ring displays a boat conformation with a dihedral angle between the pyridine rings of 43.0 (1)°. Adjacent thio­cyanate ions are linked to the cations by N—H⋯N hydrogen bonds.

## Related literature

For the crystal structures of the related cationic Pd^II^ and Pt^II^ complexes, [Pd(dpa)_2_](*X*)_2_ (*X* = Cl, PF_6_ or NO_3_) and [Pt(dpa)_2_]Br_2_·H_2_O, see: Živković *et al.* (2007 ▶); Antonioli *et al.* (2008 ▶); Ha (2012*a* ▶,*b* ▶).
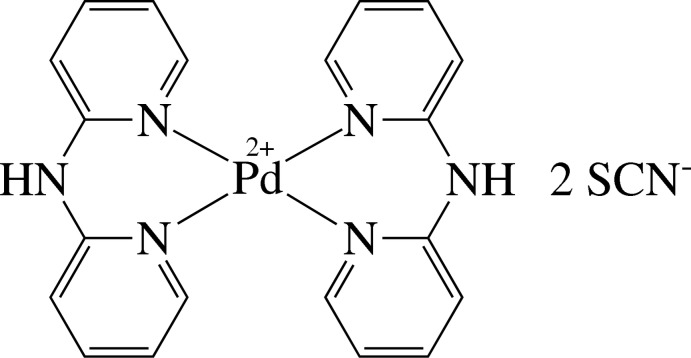


## Experimental

### 

#### Crystal data

[Pd(C_10_H_9_N_3_)_2_](NCS)_2_
*M* *_r_* = 564.96
Monoclinic, 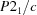

*a* = 7.7353 (9) Å
*b* = 17.478 (2) Å
*c* = 8.3822 (10) Å
β = 102.137 (2)°
*V* = 1107.9 (2) Å^3^
*Z* = 2
Mo *K*α radiation
μ = 1.06 mm^−1^
*T* = 200 K
0.16 × 0.09 × 0.09 mm

#### Data collection

Bruker SMART 1000 CCD diffractometer
Absorption correction: multi-scan (*SADABS*; Bruker, 2000 ▶) *T*_min_ = 0.876, *T*_max_ = 1.000
6810 measured reflections
2172 independent reflections
1552 reflections with *I* > 2σ(*I*)
*R*_int_ = 0.046

#### Refinement

*R*[*F* ^2^ > 2σ(*F* ^2^)] = 0.034
*wR*(*F* ^2^) = 0.088
*S* = 1.07
2172 reflections
151 parameters
H-atom parameters constrained
Δρ_max_ = 1.10 e Å^−3^
Δρ_min_ = −0.61 e Å^−3^

### 

Data collection: *SMART* (Bruker, 2000 ▶); cell refinement: *SAINT* (Bruker, 2000 ▶); data reduction: *SAINT*; program(s) used to solve structure: *SHELXS97* (Sheldrick, 2008 ▶); program(s) used to refine structure: *SHELXL97* (Sheldrick, 2008 ▶); molecular graphics: *ORTEP-3* (Farrugia, 1997 ▶) and *PLATON* (Spek, 2009 ▶); software used to prepare material for publication: *SHELXL97*.

## Supplementary Material

Crystal structure: contains datablock(s) global, I. DOI: 10.1107/S1600536812033958/ng5285sup1.cif

Structure factors: contains datablock(s) I. DOI: 10.1107/S1600536812033958/ng5285Isup2.hkl

Additional supplementary materials:  crystallographic information; 3D view; checkCIF report

## Figures and Tables

**Table 1:** Selected bond lengths (Å)

Pd1—N3	2.021 (3)
Pd1—N1	2.032 (3)

**Table 2:** Hydrogen-bond geometry (Å, °)

*D*—H⋯*A*	*D*—H	H⋯*A*	*D*⋯*A*	*D*—H⋯*A*
N2—H2*N*⋯N4^i^	0.92	1.94	2.846 (5)	170

Symmetry code: (i) 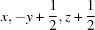.

**Fig. 1.**
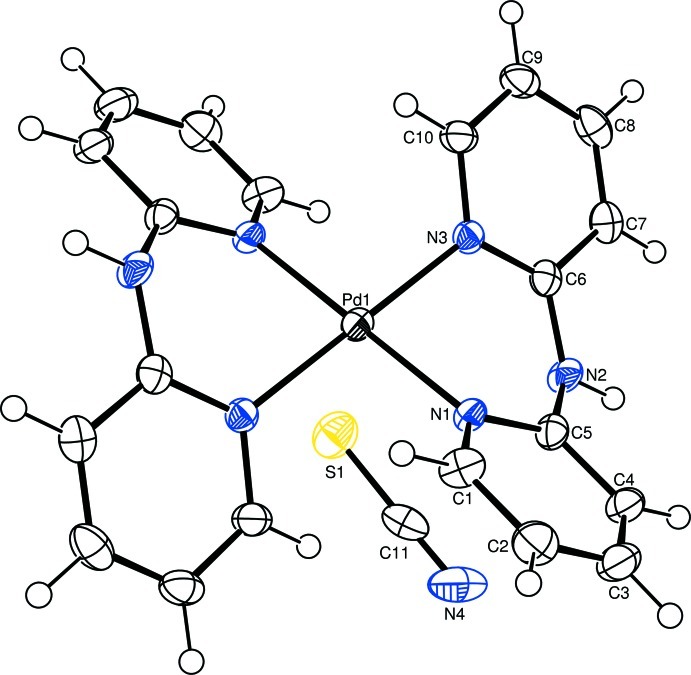
A structure detail of the title compound, with displacement ellipsoids drawn at the 50% probability level for non-H atoms. Unlabelled atoms are generated by the application of the inversion centre.

**Fig. 2.**
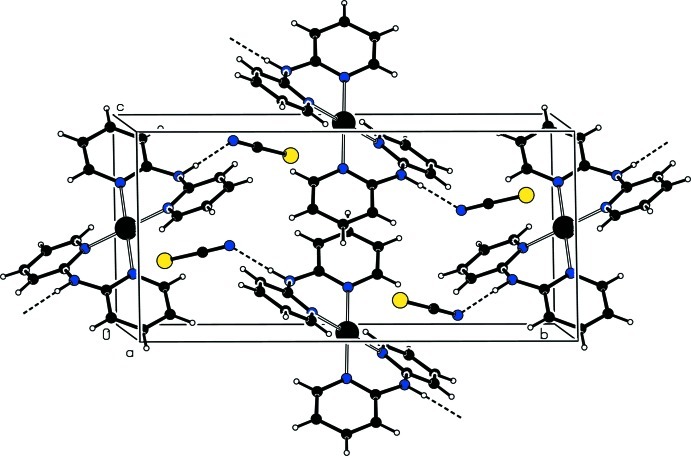
A view of the unit-cell contents of the title compound. Intermolecular N—H···N hydrogen-bond interactions are drawn with dashed lines.

## supplementary crystallographic information

### Comment

Crystal structures of the related cationic Pd^II^ and Pt^II^ complexes, such as [Pd(dpa)_2_](*X*)_2_ (dpa = di-2-pyridylamine, C_10_H_9_N_3_; *X* = Cl, PF_6_ or NO_3_) (Živković *et al.*, 2007; Antonioli *et al.*, 2008; Ha, 2012*a*) and [Pt(dpa)_2_]Br_2_.H_2_O (Ha, 2012*b*), have been investigated previously.

The asymmetric unit of the title compound, [Pd(dpa)_2_](SCN)_2_, contains one half of a cationic Pd^II^ complex and one SCN^-^ anion (Fig. 1). In the complex, the Pd^II^ ion is four-coordinated in a distorted square-planar environment by the four pyridine N atoms derived from the two chelating dpa ligands. The Pd^II^ ion is located on an inversion centre, and thus the PdN_4_ unit is exactly planar. The dpa ligands display a boat conformation with a dihedral angle between the least-squares planes of the two pyridine rings of 43.0 (1)°. The nearly planar pyridine rings [maximum deviation = 0.039 (2) Å] are considerably inclined to the PdN_4_ unit, making dihedral angles of 40.1 (2)° and 42.5 (1)°. The two Pd—N bond lengths are nearly equivalent [Pd—N: 2.021 (3) and 2.032 (3) Å] (Table 1). The SCN^-^ anion is almost linear (Table 1), and two anions are linked to the cationic complex by intermolecular N—H···N hydrogen bonds between the N atom of the anions and the N—H group of the cation (Fig. 2 and Table 2). The complex molecules are stacked into columns along the *a* axis. In the columns, several intermolecular π-π interactions between the pyridine rings are present, the shortest ring centroid-centroid distance being 3.436 (2) Å.

### Experimental

The title complex was obtained as a byproduct from the reaction of Na_2_PdCl_4_ (0.1462 g, 0.497 mmol) with KSCN (0.4688 g, 4.824 mmol) and di-2-pyridylamine (0.0877 g, 0.512 mmol) in MeOH (30 ml)/acetone (30 ml). After stirring of the reaction mixture for 24 h at room temperature, the formed precipitate was separated by filtration, washed with H_2_O and acetone, to give the main product as a pale red powder (0.1562 g). A small amount of the yellow byproduct was obtained from the mixture of filtrate and washing solution. Yellow crystals were obtained by slow evaporation from a CH_3_CN solution of the byproduct at room temperature.

### Refinement

Carbon-bound H atoms were positioned geometrically and allowed to ride on their respective parent atoms: C—H = 0.95 Å and *U*_iso_(H) = 1.2*U*_eq_(C). Nitrogen-bound H atom was located from the difference Fourier map then allowed to ride on its parent atom in the final cycles of refinement with N—H = 0.92 Å and *U*_iso_(H) = 1.5*U*_eq_(N). The highest peak (1.10 e Å^-3^) and the deepest hole (-0.61 e Å^-3^) in the difference Fourier map are located 0.85 Å and 1.48 Å, respectively, from the atoms C1 and H1.

### Crystal data

**Table:**

[Pd(C_10_H_9_N_3_)_2_](NCS)_2_	*F*(000) = 568
*M**_r_* = 564.96	*D*_x_ = 1.694 Mg m^−^^3^
Monoclinic, *P*2_1_/*c*	Mo *K*α radiation, λ = 0.71073 Å
Hall symbol: -P 2ybc	Cell parameters from 2711 reflections
*a* = 7.7353 (9) Å	θ = 2.3–25.9°
*b* = 17.478 (2) Å	µ = 1.06 mm^−^^1^
*c* = 8.3822 (10) Å	*T* = 200 K
β = 102.137 (2)°	Block, yellow
*V* = 1107.9 (2) Å^3^	0.16 × 0.09 × 0.09 mm
*Z* = 2	

### Data collection

**Table:**

Bruker SMART 1000 CCD diffractometer	2172 independent reflections
Radiation source: fine-focus sealed tube	1552 reflections with *I* > 2σ(*I*)
Graphite monochromator	*R*_int_ = 0.046
φ and ω scans	θ_max_ = 26.0°, θ_min_ = 2.3°
Absorption correction: multi-scan (*SADABS*; Bruker, 2000)	*h* = −9→6
*T*_min_ = 0.876, *T*_max_ = 1.000	*k* = −21→20
6810 measured reflections	*l* = −10→10

### Refinement

**Table:**

Refinement on *F*^2^	Primary atom site location: structure-invariant direct methods
Least-squares matrix: full	Secondary atom site location: difference Fourier map
*R*[*F*^2^ > 2σ(*F*^2^)] = 0.034	Hydrogen site location: inferred from neighbouring sites
*wR*(*F*^2^) = 0.088	H-atom parameters constrained
*S* = 1.07	*w* = 1/[σ^2^(*F*_o_^2^) + (0.0333*P*)^2^ + 0.3195*P*] where *P* = (*F*_o_^2^ + 2*F*_c_^2^)/3
2172 reflections	(Δ/σ)_max_ < 0.001
151 parameters	Δρ_max_ = 1.10 e Å^−^^3^
0 restraints	Δρ_min_ = −0.61 e Å^−^^3^

### Special details

**Table:**

Geometry. All e.s.d.'s (except the e.s.d. in the dihedral angle between two l.s. planes) are estimated using the full covariance matrix. The cell e.s.d.'s are taken into account individually in the estimation of e.s.d.'s in distances, angles and torsion angles; correlations between e.s.d.'s in cell parameters are only used when they are defined by crystal symmetry. An approximate (isotropic) treatment of cell e.s.d.'s is used for estimating e.s.d.'s involving l.s. planes.
Refinement. Refinement of *F*^2^ against ALL reflections. The weighted *R*-factor *wR* and goodness of fit *S* are based on *F*^2^, conventional *R*-factors *R* are based on *F*, with *F* set to zero for negative *F*^2^. The threshold expression of *F*^2^ > σ(*F*^2^) is used only for calculating *R*-factors(gt) *etc*. and is not relevant to the choice of reflections for refinement. *R*-factors based on *F*^2^ are statistically about twice as large as those based on *F*, and *R*- factors based on ALL data will be even larger.

### Fractional atomic coordinates and isotropic or equivalent isotropic displacement parameters (Å^2^)

**Table:**

	*x*	*y*	*z*	*U*_iso_*/*U*_eq_	
Pd1	0.5000	0.0000	0.5000	0.02072 (15)	
N1	0.6536 (4)	0.08760 (17)	0.6087 (4)	0.0216 (7)	
N2	0.4264 (4)	0.12533 (17)	0.7419 (4)	0.0240 (8)	
H2N	0.3803	0.1669	0.7859	0.036*	
N3	0.4123 (4)	−0.00805 (17)	0.7101 (4)	0.0199 (7)	
C1	0.8191 (5)	0.0992 (2)	0.5843 (5)	0.0274 (9)	
H1	0.8688	0.0616	0.5253	0.033*	
C2	0.9181 (5)	0.1620 (2)	0.6399 (5)	0.0319 (10)	
H2	1.0339	0.1681	0.6204	0.038*	
C3	0.8453 (6)	0.2168 (2)	0.7258 (5)	0.0329 (11)	
H3	0.9097	0.2619	0.7633	0.039*	
C4	0.6808 (5)	0.2056 (2)	0.7562 (5)	0.0283 (10)	
H4	0.6303	0.2427	0.8155	0.034*	
C5	0.5868 (5)	0.1392 (2)	0.6995 (4)	0.0223 (9)	
C6	0.3700 (5)	0.0553 (2)	0.7841 (4)	0.0207 (9)	
C7	0.2737 (5)	0.0502 (2)	0.9079 (5)	0.0283 (10)	
H7	0.2371	0.0954	0.9545	0.034*	
C8	0.2331 (5)	−0.0194 (2)	0.9605 (5)	0.0303 (11)	
H8	0.1663	−0.0234	1.0432	0.036*	
C9	0.2900 (5)	−0.0854 (2)	0.8927 (5)	0.0293 (10)	
H9	0.2673	−0.1346	0.9315	0.035*	
C10	0.3784 (5)	−0.0773 (2)	0.7701 (5)	0.0247 (9)	
H10	0.4184	−0.1220	0.7244	0.030*	
S1	0.15609 (16)	0.10410 (7)	0.31675 (16)	0.0421 (3)	
N4	0.3250 (5)	0.2438 (2)	0.4024 (5)	0.0511 (11)	
C11	0.2561 (6)	0.1855 (3)	0.3654 (5)	0.0326 (10)	

### Atomic displacement parameters (Å^2^)

**Table:**

	*U*^11^	*U*^22^	*U*^33^	*U*^12^	*U*^13^	*U*^23^
Pd1	0.0234 (3)	0.0179 (2)	0.0213 (2)	−0.00227 (18)	0.00572 (16)	−0.00236 (19)
N1	0.0248 (19)	0.0182 (18)	0.0214 (18)	−0.0019 (13)	0.0037 (14)	−0.0016 (14)
N2	0.0285 (19)	0.0178 (18)	0.0275 (19)	0.0043 (14)	0.0099 (15)	−0.0034 (14)
N3	0.0189 (17)	0.0213 (18)	0.0186 (16)	−0.0031 (13)	0.0023 (13)	−0.0019 (14)
C1	0.019 (2)	0.025 (2)	0.040 (3)	0.0017 (17)	0.0094 (18)	0.0015 (19)
C2	0.024 (2)	0.040 (3)	0.031 (2)	−0.0078 (19)	0.0037 (19)	0.003 (2)
C3	0.038 (3)	0.023 (2)	0.034 (3)	−0.0071 (19)	−0.001 (2)	0.0007 (19)
C4	0.035 (3)	0.019 (2)	0.029 (2)	−0.0023 (17)	0.0043 (19)	−0.0002 (18)
C5	0.024 (2)	0.021 (2)	0.019 (2)	0.0034 (16)	−0.0014 (16)	0.0011 (17)
C6	0.019 (2)	0.024 (2)	0.017 (2)	0.0009 (16)	0.0000 (16)	−0.0010 (17)
C7	0.025 (2)	0.034 (3)	0.026 (2)	0.0012 (18)	0.0031 (18)	−0.0058 (19)
C8	0.021 (2)	0.049 (3)	0.022 (2)	−0.0057 (18)	0.0057 (17)	0.0035 (19)
C9	0.028 (2)	0.030 (2)	0.026 (2)	−0.0100 (18)	−0.0002 (18)	0.0047 (19)
C10	0.025 (2)	0.024 (2)	0.024 (2)	−0.0045 (17)	0.0036 (18)	0.0033 (18)
S1	0.0391 (7)	0.0371 (7)	0.0512 (8)	0.0015 (5)	0.0122 (6)	−0.0057 (6)
N4	0.053 (3)	0.041 (3)	0.060 (3)	−0.011 (2)	0.013 (2)	0.018 (2)
C11	0.029 (3)	0.039 (3)	0.031 (3)	0.008 (2)	0.010 (2)	0.013 (2)

### Geometric parameters (Å, º)

**Table:**

Pd1—N3	2.021 (3)	C3—C4	1.363 (5)
Pd1—N3^i^	2.021 (3)	C3—H3	0.9500
Pd1—N1	2.032 (3)	C4—C5	1.399 (5)
Pd1—N1^i^	2.032 (3)	C4—H4	0.9500
N1—C5	1.350 (5)	C6—C7	1.402 (5)
N1—C1	1.354 (5)	C7—C8	1.355 (6)
N2—C6	1.371 (5)	C7—H7	0.9500
N2—C5	1.382 (5)	C8—C9	1.396 (6)
N2—H2N	0.9200	C8—H8	0.9500
N3—C6	1.342 (5)	C9—C10	1.356 (5)
N3—C10	1.358 (5)	C9—H9	0.9500
C1—C2	1.364 (5)	C10—H10	0.9500
C1—H1	0.9500	S1—C11	1.630 (5)
C2—C3	1.387 (6)	N4—C11	1.161 (5)
C2—H2	0.9500		
			
N3—Pd1—N3^i^	180.0	C2—C3—H3	120.2
N3—Pd1—N1	86.18 (12)	C3—C4—C5	119.6 (4)
N3^i^—Pd1—N1	93.82 (12)	C3—C4—H4	120.2
N3—Pd1—N1^i^	93.82 (12)	C5—C4—H4	120.2
N3^i^—Pd1—N1^i^	86.18 (12)	N1—C5—N2	119.9 (3)
N1—Pd1—N1^i^	180.00 (11)	N1—C5—C4	120.8 (4)
C5—N1—C1	118.1 (3)	N2—C5—C4	119.2 (3)
C5—N1—Pd1	119.9 (3)	N3—C6—N2	119.7 (3)
C1—N1—Pd1	121.8 (3)	N3—C6—C7	120.6 (4)
C6—N2—C5	125.0 (3)	N2—C6—C7	119.6 (4)
C6—N2—H2N	115.5	C8—C7—C6	119.6 (4)
C5—N2—H2N	114.2	C8—C7—H7	120.2
C6—N3—C10	118.6 (3)	C6—C7—H7	120.2
C6—N3—Pd1	120.3 (2)	C7—C8—C9	119.6 (4)
C10—N3—Pd1	120.8 (3)	C7—C8—H8	120.2
N1—C1—C2	123.3 (4)	C9—C8—H8	120.2
N1—C1—H1	118.3	C10—C9—C8	118.4 (4)
C2—C1—H1	118.3	C10—C9—H9	120.8
C1—C2—C3	118.2 (4)	C8—C9—H9	120.8
C1—C2—H2	120.9	C9—C10—N3	122.7 (4)
C3—C2—H2	120.9	C9—C10—H10	118.6
C4—C3—C2	119.7 (4)	N3—C10—H10	118.6
C4—C3—H3	120.2	N4—C11—S1	178.6 (4)
			
N3—Pd1—N1—C5	−41.3 (3)	C6—N2—C5—N1	38.3 (5)
N3^i^—Pd1—N1—C5	138.7 (3)	C6—N2—C5—C4	−139.6 (4)
N3—Pd1—N1—C1	143.2 (3)	C3—C4—C5—N1	−3.0 (6)
N3^i^—Pd1—N1—C1	−36.8 (3)	C3—C4—C5—N2	174.9 (3)
N1—Pd1—N3—C6	44.1 (3)	C10—N3—C6—N2	170.2 (3)
N1^i^—Pd1—N3—C6	−135.9 (3)	Pd1—N3—C6—N2	−16.2 (4)
N1—Pd1—N3—C10	−142.4 (3)	C10—N3—C6—C7	−7.7 (5)
N1^i^—Pd1—N3—C10	37.6 (3)	Pd1—N3—C6—C7	165.9 (3)
C5—N1—C1—C2	−3.3 (6)	C5—N2—C6—N3	−35.6 (5)
Pd1—N1—C1—C2	172.3 (3)	C5—N2—C6—C7	142.3 (4)
N1—C1—C2—C3	0.0 (6)	N3—C6—C7—C8	4.4 (5)
C1—C2—C3—C4	1.9 (6)	N2—C6—C7—C8	−173.5 (3)
C2—C3—C4—C5	−0.4 (6)	C6—C7—C8—C9	1.0 (6)
C1—N1—C5—N2	−173.1 (3)	C7—C8—C9—C10	−2.8 (6)
Pd1—N1—C5—N2	11.2 (5)	C8—C9—C10—N3	−0.6 (6)
C1—N1—C5—C4	4.7 (5)	C6—N3—C10—C9	5.9 (5)
Pd1—N1—C5—C4	−170.9 (3)	Pd1—N3—C10—C9	−167.7 (3)

Symmetry code: (i) −*x*+1, −*y*, −*z*+1.

### Hydrogen-bond geometry (Å, º)

**Table:**

*D*—H···*A*	*D*—H	H···*A*	*D*···*A*	*D*—H···*A*
N2—H2*N*···N4^ii^	0.92	1.94	2.846 (5)	170

Symmetry code: (ii) *x*, −*y*+1/2, *z*+1/2.
